# Effects of miR-185-5p on replication of hepatitis C virus

**DOI:** 10.1515/biol-2021-0067

**Published:** 2021-08-02

**Authors:** Wei Huang, Lingyan Song, Jingyan Zhang, Xueqiang Yan, Hui Yan

**Affiliations:** Department of Laboratory Medicine, Heping Hospital Affiliated to Changzhi Medical College, No. 110 Yan’an Nan Road, Changzhi 046000, China

**Keywords:** hepatitis C virus, replication, miR-185-5p, GALNT8

## Abstract

This article was designed to explore the effects and mechanisms of miR-185-5p on the replication of hepatitis C virus (HCV). Quantitative reverse transcription PCR (qRT-PCR) was performed for detecting the abundance of miR-185-5p and HCV RNA in HCV-infected primary hepatocytes and Huh7.5 cells. Dual-luciferase reporter gene assay was used for exploring the interaction between miR-185-5p and GALNT8. Western blot analyzed protein expression of GALNT8, NS3, and NS5A. miR-185-5p was remarkably downregulated in HCV-infected primary hepatocytes and Huh7.5 cells. miR-185-5p upregulation inhibited HCV RNA expression, while its inhibition promoted HCV replication. miR-185-5p induced accumulation of NS3 and NS5A in the cells. Dual-luciferase reporter gene assay verified the targeted relationship between miR-185-5p and GALNT8. In addition, the effects of overexpressing or knocking down miR-185-5p on HCV replication could be correspondingly eliminated by the overexpression or knockdown of GALNT8. miR-185-5p may target GALNT8 in JFH1-infected Huh7.5 cells and then inhibit HCV replication. miR-185-5p may be a potential target for treating HCV.

## 1 Introduction

Hepatitis C virus (HCV), belonging to Flaviviridae and including one single open reading frame, is a RNA virus (single positive-strand) with envelopes, with its frame-encoding polyproteins of 3,000 amino acids [1,2]. After translation, polyproteins are processed into viral structural proteins and nonstructural proteins (NS3, NS5A) [3]. HCV core protein, considered to be a pathogenic factor, induces fatty degeneration, hepatocellular carcinoma, and oxidative stress [4]. However, currently, there is no effective vaccine to prevent HCV infection, which is mainly treated by ribavirin-based antiviral therapy in clinical practice, and only some patients can produce sustained immune responses [5,6].

As small noncoding RNAs (single stranded, about 22 nucleotides long), microRNAs (miRNAs) can inhibit the expression of target genes by imperfectly pairing with miRNA response elements in 3′-untranslated regions (UTRs) [7]. As one of them, miR-185-5p is mainly considered a regulatory factor for cancer progression [8]. Based on the latest research, miR-185-5p is involved in how HCV core protein regulates SREBP2 [9]. As reported by previous studies, HCV can induce the fatty degeneration of the liver to enhance its replication [10], and the host’s cholesterol metabolism is essential for the complete life cycle of HCV [11]. Accordingly, miR-185-5p may have a regulatory effect on HCV replication, which has been rarely studied.

Herein, we explored the effects of miR-185-5p on HCV replication and the possible mechanisms. Our findings may provide potential therapeutic targets for HCV treatment.

## 2 Materials and methods

### 2.1 Cell culture

Primary human hepatocytes and human Huh7.5 cells were purchased from ATCC. By using MEGAscript RNAi kits, HCV genotype 2a (JFH1) RNA was prepared and then seeded into the cells with Lipofectamine 2000. The cells were cultured in DMEM medium supplemented with 10% fetal bovine serum (a humidified incubator, 5% CO_2_, 37°C).

### 2.2 Cell transfection

For upregulating or inhibiting miR-185-5p expression, miR-185-5p mimics (miR-185-5p) or miR-185-5p inhibitor (anti-miR-185-5p) and its negative control (miR-NC) were transfected into Huh7.5 cells. For overexpressing GALNT8, pcDNA3.1-GALNT8 (GALNT8) was introduced into Huh7.5.1 cells, and pcDNA3.1 empty vector (Vector) was used as a control. For downregulating GALNT8, siRNA targeting GALNT8 (si-GALNT8) was transfected into the cells and siRNA (si-NC) was also used as a control. According to the manufacturer’s instruction, Lipofectamine 2000 was applied to transfection.

### 2.3 Reverse transcription quantitative PCR (RT-qPCR)

With reference to the TRIzol reagents manufacturer’s instruction, total RNA was extracted from the cells, and then, the NanoDrop spectrophotometer was adopted to determine its concentration and purity. Then, based on the instruction of reverse transcription kits, the mRNA or the miRNA was used for the synthesis of the first-strand cDNA. The quantitative reverse transcription PCR (qRT-PCR) was performed with SYBR Green. The amplification steps were as follows: 95°C for 60 s, 95°C for 15 s, and 60°C for 1 min, which were cycled 40 times. The 2^−ΔΔCt^ was used for analyzing the results, with β-Actin and U6 as control genes. The following primers (Invitrogen) were designed: forward and reverse primers for HCV were 5′-TCTGCGGAACCGGTGAGTA-3′ and 5′-TCAGGCACTACCACAAGGC-3′, respectively. Those for β-Actin were 5′-AGCAGCATCGCCCCAAAGTT-3′ and 5′-GGGCACGAAGGCTCATCATT-3′, respectively. Those for miR-185-5p were 5′-CGCTGGAGAGAAAGGCAGT-3′ and 5′-GTGCAGGGTCCGAGGT-3′, respectively. Those for U6 were 5′-CTCGCTTCGGCAGCACA-3′ and 5′ -AACGCTTCACGAATTTGCGT-3′, respectively. All measurements were repeated in triplicate.

### 2.4 Western blot

The cultured cells in each group were collected and subjected to RIPA (Thermo Scientific, USA) lysis for extracting the total protein. The BCA (Thermo Scientific, USA) method was used to detect its concentration. After the protein concentration was adjusted to 4 μg/μL, separation with 12% SDS-PAGE was performed. Subsequently, proteins were transferred to a PVDF membrane. Next, Ponceau S working solution was used for staining the membrane, which was immersed in PBST (5 min). After being cleaned and sealed (2 h) with 5% skimmed milk, the membrane was sealed (4°C) overnight with GALNT8 (1:500), NS3 (1:500), NS5A (1:500), and β-actin (1:500; Cell Signaling Technology). After being washed to remove the primary antibodies, a horseradish peroxidase-labeled secondary antibody (goat anti-rabbit (1:1,000)) was added, and the membranes were incubated at 37°C for 1 h, and rinsed with PBS (5 min) three times. Finally, it was developed with the ECL luminescence reagent. The gray values were analyzed.

### 2.5 Dual-luciferase reporter gene assay (DLRGA)

Dual-luciferase reporter plasmids (WT and MUT) of GALNT8 3′-UTR were constructed through RiboBio and co-transfected with miR-185-5p or miR-NC into the cells using Lipofectamine 2000. After 48 h, based on the manufacturer’s instruction, luciferase activities were detected through the dual-luciferase reporter assay system.

### 2.6 Statistical analysis

In this study, SPSS 19.0 was adopted for data analysis and GraphPad Prism 6 for plotting relevant figures. Results were expressed as mean ± standard deviation (SD ± means), and measurement data were compared by the *t* test. The comparison between groups was conducted by the independent samples *t* test and represented by *t*. The comparison between multiple groups was conducted by one-way analysis of variance, and the LSD-*t* test was used for *post hoc* pairwise comparison. Bonferroni was applied to the *post hoc* test, and Pearson was applied to the correlation analysis. When *P* < 0.05, the difference was statistically significant.

## 3 Results

### 3.1 HCV infection could inhibit miR-185-5p in hepatocytes

**Figure 1:**
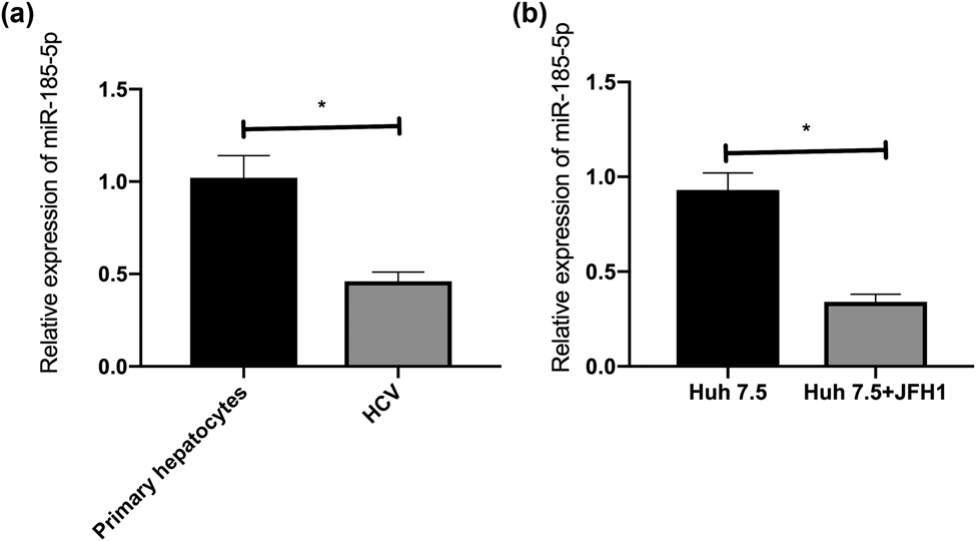
HCV infection led to miR-185-5p downregulation. (a) miR-185-5p expression in HCV-infected primary hepatocytes. (b) miR-185-5p expression in HCV-infected Huh7.5 cells. *P < 0.05.

Compared with untreated primary hepatocytes, the expression of miR-185-5p was downregulated in HCV-infected primary human hepatocytes (Figure 1a). Compared with Huh7.5 cells, the expression of miR-185-5p was downregulated in Huh7.5 cells transfected with JFH1 (Figure 1b). Accordingly, HCV infection could inhibit miR-185-5p in hepatocytes.

### 3.2 miR-185-5p inhibited HCV replication

**Figure 2:**
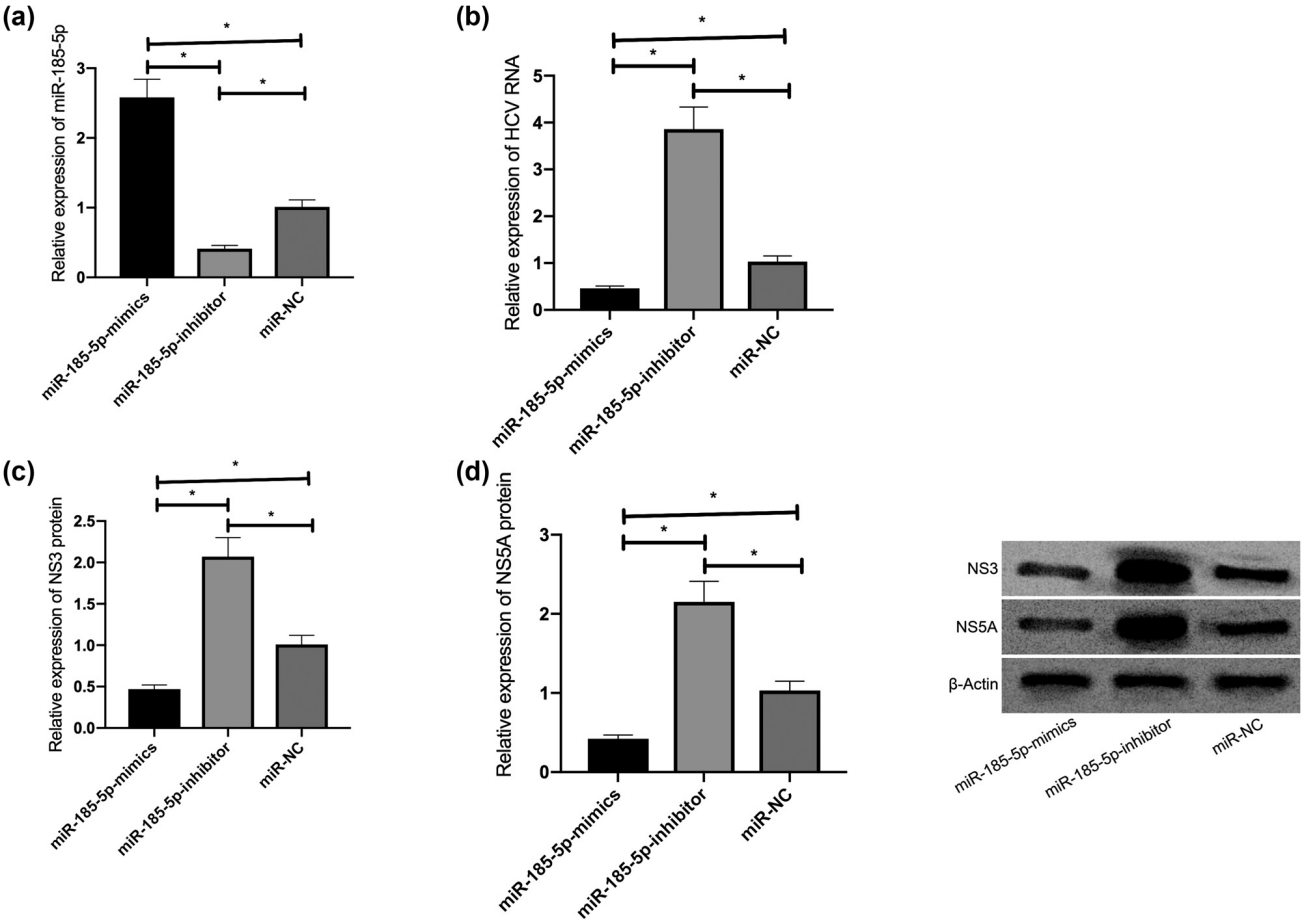
miR-185-5p inhibited HCV replication. (a) The transfection efficiency of miR-185-5p in Huh7.5 cells. (b) The effects of miR-185-5p on HCV RNA replication in cells. (c) The effects of miR-185-5p on NS3 in cells. (d) The effects of miR-185-5p on NS5A in cells. *P < 0.05.

To determine the effects of miR-185-5p on HCV replication, miR-185-5p or anti-miR-185-5p was transfected into JFH1-infected Huh7.5 cells, and transfection efficiency was confirmed by the qRT-PCR. Among them, miR-185-5p was overexpressed by its mimics and inhibited by its inhibitor successfully (Figure 2a). In addition, in JFH1-infected Huh7.5 cells, the overexpression of miR-185-5p inhibited HCV RNA expression, while the inhibition of miR-185-5p promoted HCV RNA expression (Figure 2b). According to the western blot, the miR-185-5p overexpression inhibited the accumulation of NS3 and NS5A in the cells (Figure 2c and d), suggesting that miR-185-5p can inhibit HCV replication while inhibiting miR-185-5p has an opposite effect. Accordingly, miR-185-5p is involved in the progression of HCV infection.

### 3.3 DLRGA

**Figure 3:**
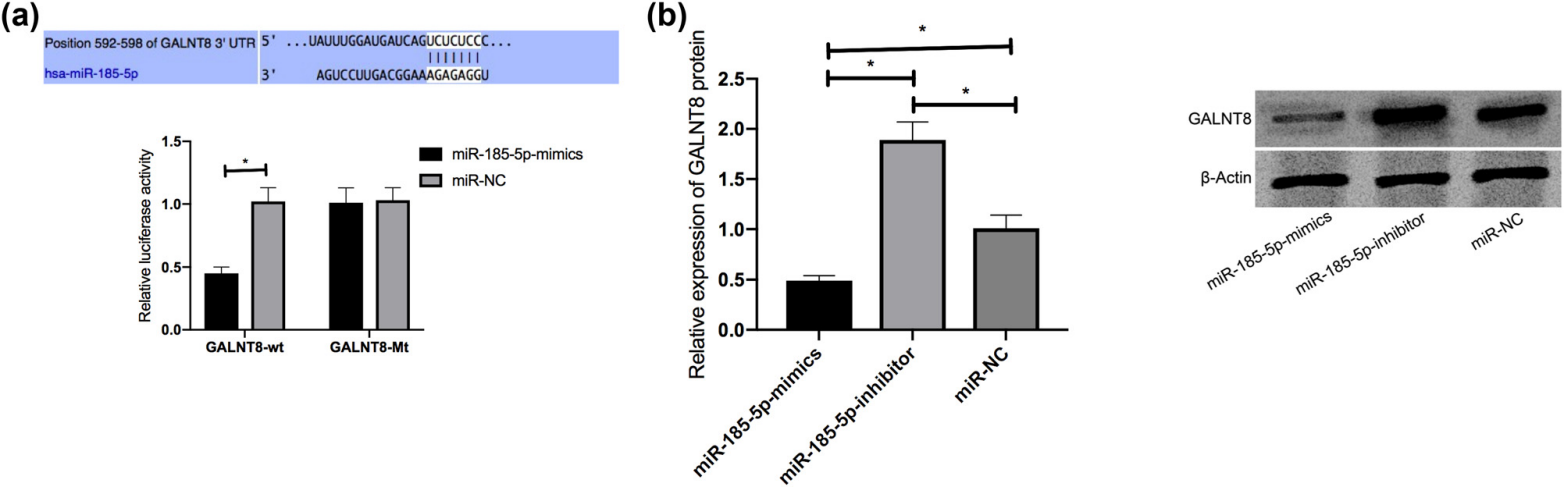
DLRGA. (a) The effects of miR-185-5p on luciferase activities of GALNT8. (b) The effects of miR-185-5p on GALNT8 in Huh7.5 cells. *P < 0.05.

After confirming that miR-185-5p can inhibit HCV replication, we tried to determine whether this miR interacts with GALNT8. According to the bioinformatics analysis, the online tool TargetScan predicted the putative binding sites between the two, which indicates that GALNT8 may be the direct target gene of miR-185-5p (Figure 3a). DLRGA confirmed that miR-185-5p directly targeted GALNT8 (Figure 3a). When this miR was overexpressed in Huh7.5 cells, the luciferase activity of GALNT8-wt declined remarkably. Western blot was also performed to detect GALNT8 in the JFH1-infected Huh7.5 cells transfected with miR-185-5p or anti-miR-185-5p. miR-185-5p overexpression could inhibit GALNT8 expression, while the inhibition of this miR could enhance the expression (Figure 3b).

### 3.4 GALNT8 could reverse the inhibitory effect of miR-185-5p on HCV replication

**Figure 4:**
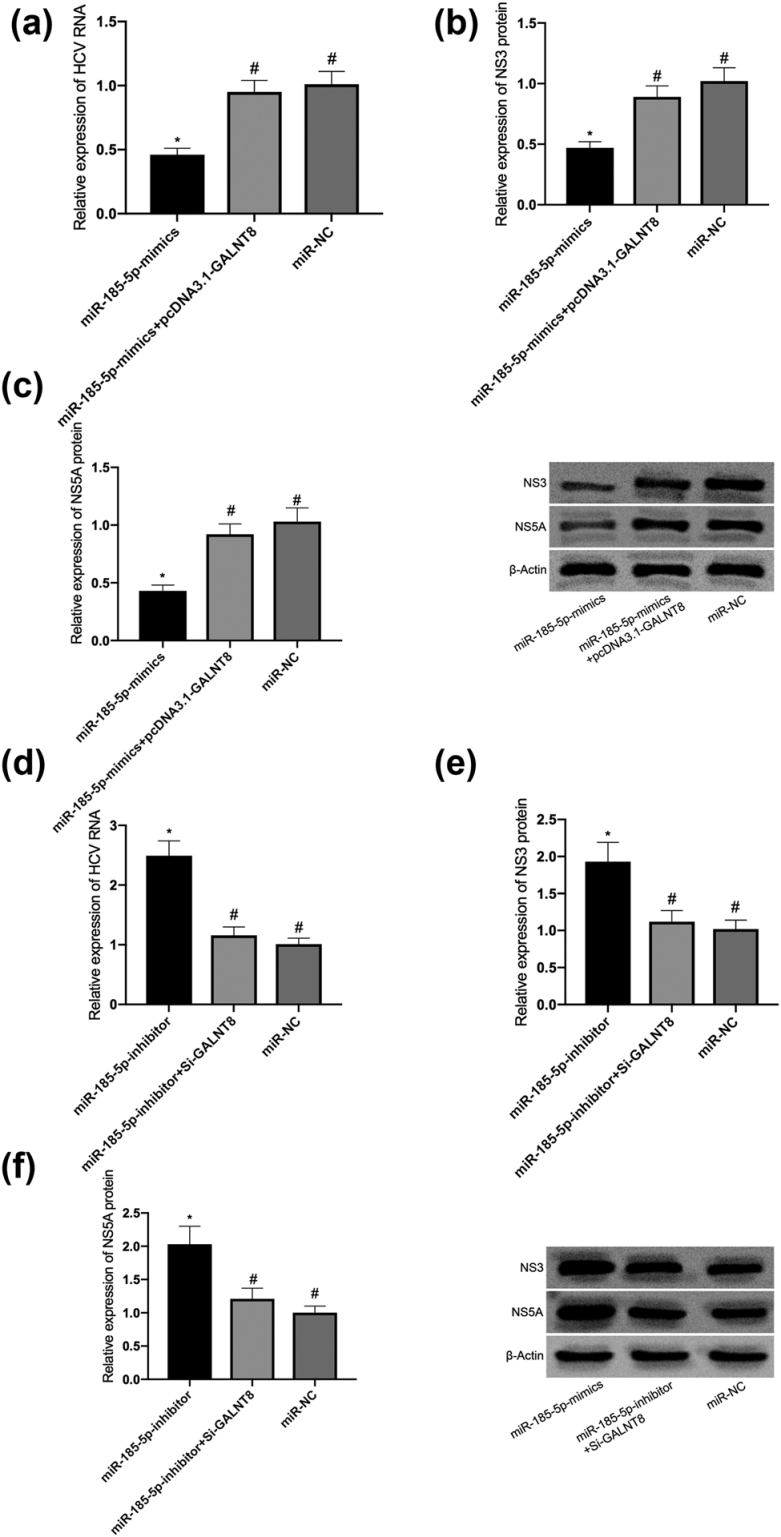
GALNT8 could reverse the inhibitory effect of miR-185-5p on HCV replication. (a) The effects of pcDNA3.1-GALNT8 on HCV RNA replication after miR-185-5p transfection. (b) The effects of pcDNA3.1-GALNT8 on NS3 after miR-185-5p transfection. (c) The effects of pcDNA3.1-GALNT8 on NS5A after miR-185-5p transfection. (d) The effects of si-GALNT8 on HCV RNA replication after anti-miR-185-5p transfection. (e) The effects of si-GALNT8 on NS3 after anti-miR-185-5p transfection. (f) The effects of si-GALNT8 on NS5A after anti-miR-185-5p transfection. * was compared with ^#^, *P* < 0.05.

For further exploring whether GALNT8 could eliminate the inhibitory effect of miR-185-5p on HCV replication, miR-185-5p, miR-185-5p + pcDNA3.1-GALNT8, and anti-miR-185-5p or anti-miR-185-5p + si-GALNT8 were transfected into JFH1-infected Huh7.5 cells. Compared with miR-185-5p alone, the addition of GALNT8 remarkably enhanced HCV replication in the cells (Figure 4a). On the contrary, the depletion of GALNT8 reversed the effects, which were mediated by anti-miR-185-5p, on promoting HCV replication, thereby reducing HCV RNA (Figure 4d). Compared with the pcDNA treatment group, GALNT8 could remarkably increase NS3 and NS5A in the cells transfected with miR-185-5p (Figure 4b and c), while inhibiting this protein had an opposite effect (Figure 4e and f). These findings suggest that GALNT8 can reverse the inhibitory effect of miR-185-5p on HCV replication in JFH1-infected Huh7.5 cells.

## 4 Discussion

In recent years, there has been more evidence that miRNAs exert a significant function in regulating HCV pathogenesis [12]. It has been previously reported that miR-122 is a promising candidate for anti-HCV therapy because its inhibitory effect reduces the richness of HCV RNA [13,14]. In this study, we have confirmed that miR-185-5p inhibits HCV replication by targeting GALNT8.

According to previous studies, miR-185-5p has a significant effect on virus-related liver cancer. For instance, it can reduce the activity of HBV S1p by targeting ELK1, thus inhibiting the progression of HBV-related liver cancer [15]. In our study, miR-185-5p was remarkably reduced in HCV-infected human liver cells and liver cancer cells, suggesting that the miR might be related to the activities of HCV viruses. Some researchers identified miRNA expression profiles in the serum of patients with HCV-related hepatocellular carcinoma, finding that miR-185-5p remarkably declined in the serum [16]. That is consistent with our results. Then, for observing influences of miR-185-5p on HCV replication, we overexpressed and inhibited it in JFH1-infected Huh7.5 cells because JFH1 is the first HCV strain producing HCV particles and has a significant effect on the progression of HCV [17]. Subsequently, we observed that the overexpression could remarkably inhibit the richness of HCV, but the inhibition could promote HCV replication, which was confirmed by the increase in HCV RNA and NS3 and NS5A levels. According to a previous study, miR-185-5p can promote HCV replication [18], in which our experiments have further revealed the role of miRNAs.

Next, we further discussed the possible molecular basis of HCV replication induced by miR-185-5p. According to the bioinformatics analysis and the DLRGA, GALNT8 was identified as the direct target of miR-185-5p. As we all know, miRNA has multiple targets. According to relevant references, GALNT8 is a miR-185-5p target related to HCV replication, so we chose GALNT8 as the research target. As a member of the O-linked UDP-*N*-acetylglucosamine (GalNAc) glycosyltransferase family, GALNT8 transfers GalNAc to serine and threonine residues on target proteins in the Golgi apparatus, thus taking part in the biosynthesis of mucin-type O-glycans [19,20].

Moreover, single nucleotide polymorphism in GALNT8 is related to the responses of HCV to interferon therapy [21]. In our study, knocking down GALNT8 could inhibit HCV replication in Huh7.5 cells, which was manifested by the decrease in HCV RNA and levels of NS3 and NS5A. In addition, the miR-185-5p-induced inhibition of HCV replication was destroyed by GALNT8 upregulation in Huh7.5 cells, which suggests that miR-185-5p inhibits HCV replication by inhibiting GALNT8. The results of the rescue experiment also confirmed that miR-185-5p inhibited HCV replication through a specific molecular mechanism, and this study has initially verified the targeted relationship between the two markers in HCV infection.

## 5 Conclusion

To sum up, in this study, first, the interaction between miR-185-5p and HCV infection has been primarily explored based on fundamental research. HCV infection inhibits miR-185-5p, and overexpressing the miR inhibits the replication by inhibiting GALNT8 in JFH1-infected Huh7.5 cells. This demonstrates that this miR has a potential targeting effect and is conducive to formulating targeted therapeutic schemes for HCV infection. However, this study also has some limitations. For example, we have not yet verified the effect of miR-185-5p on HCV replication *in vivo*. Second, the downstream pathway mechanism of GALNT8 still needs to be further explored. However, this study has provided a certain experimental basis for inhibiting HCV replication based on a primary research, and we will conduct further research on this to get more data support for finding possible targets for inhibiting HCV.
